# Genomic and Expression Analysis of Cassava (*Manihot esculenta* Crantz) Chalcone Synthase Genes in Defense against *Tetranychus cinnabarinus* Infestation

**DOI:** 10.3390/genes15030336

**Published:** 2024-03-05

**Authors:** Yanni Yang, Ming Liu, Zenghui Huang

**Affiliations:** 1 Guangxi Key Laboratory of Plant Functional Phytochemicals and Sustainable Utilization, Guangxi Institute of Botany, Guangxi Zhuang Autonomous Region and Chinese Academy of Sciences, Guilin 541006, China; liuming@gxib.cn; 2 College of Agronomy, Guangxi University, Nanning 530004, China; 3 Nanning New Technology Entrepreneur Center, Nanning 530007, China; nnxjscyzzx2023@163.com

**Keywords:** CHS genes family, *Manihot esculenta* Crantz, *Tetranychus cinnabarinus*, expression patterns

## Abstract

Cassava is susceptible to mites, especially *Tetranychus cinnabarinus*. Secondary metabolism products such as flavonoids play an important role as antimicrobial metabolites protecting plants against biotic stressors including fungal, pathogen, bacterial, and pest defense. The chalcone synthase (CHS) is the initial step of the phenylpropanoid pathway for producing flavonoids and is the gatekeeper of the pathway. Until recently, the CHS genes family has not been systematically studied in cassava. Thirty-nine CHS genes were identified from the cassava genome database. Based on phylogenetic and sequence composition analysis, these CHSs were divided into 3 subfamilies. Within the same subfamily, the gene structure and motif compositions of these CHS genes were found to be quite conserved. Duplication events, particularly segmental duplication of the cassava CHS genes, were identified as one of the main driving force of its expansion. Various cis-elements contained in the promoter might regulate the gene expression patterns of *MeCHS*. Protein-protein interaction (PPI) network analysis showed that *MeCHS*1 and *MeCHS*10 protein are more closely related to other family members. The expression of *MeCHS* genes in young leaves was higher than that in other tissues, and their expression varies even within the same tissue. Coincidentally, these CHS genes of most LAP subclasses were highly expressed in young leaves. The verified *MeCHS* genes showed consistent with the real-time reverse transcription quantitative PCR (RT-qPCR) and proteomic expression in protected and affected leaves respectively, indicating that these *MeCHS* genes play crucial roles in the response to *T. cinnabarinus*. This study is the first to comprehensively expatiate the information on *MeCHS* family members. These data will further enhance our understanding both the molecular mechanisms and the effects of CHS genes. In addition, the results will help to further clarify the effects on *T. cinnabarinus* and provide a theoretical basis for the potential functions of the specific CHS gene in resistance to mites and other biotic stress.

## 1. Introduction

The chalcone synthase (CHS) is a well-known initial enzyme for catalyzing the formation of secondary metabolism products such as flavonoids, isoflavonoids and anthocyanin in the phenylpropanoid pathway, which are widely distributed in plant kingdom. Indeed, CHSs are member of the plant-specific type III polyketide synthase (PKS) family [1,2]. CHS, the first type III plant PKS enzyme to be discovered, has a primary role in the biosynthesis of flavonoids, which is encoded by a multigene [3,4,5]. 

Flavonoids not only have a wide variety of biological functions in flower pigmentation, protection against UV radiation [6,7,8], stress resistance during growth and development, auxin transport and pollen fertility [9,10,11,12,13], but also play an important and respective role as antimicrobial metabolites in protecting plants against biotic stressors including fungal, pathogen, bacterial, and pest defense [14]. Since 1983, when the first complete cDNA copy of CHS mRNA was isolated from cultured parsley cells (*Petroselinum hortense*), nearly 20 functionally distinct CHS superfamilies have been identified and further characterized [15,16]. According to the topology and architecture of CHSs studied, a set of 32 amino acids residues were proposed to participate in the formation of the active site pocket, the binding of coenzyme A to the initiator group, catalysis, cyclization and other relevant events in the enzyme process [17]. CHS has been shown to a significant correlation with synthesis of flavonoid compounds during response against biotic factors including fungal, pathogen, bacterial, and pest defense which in turn causes promote the production of flavonoids [14,18,19,20,21]. The expression level of CHS in *Phaseolus vulgaris* was significantly increased after 20 min treatment with *Colletotrichum lindemuthianum* extract, and peaked at 2.5 h after treatment, indicating that the CHS gene was induced by the activator in the extract [22]. Ran Liu et al. found that the emergence of CHSV and CHSVII, which contain an N-terminal myosin motor-like domain (MMD, PF00063), are important for the development of fungal morphogenesis [23]. It was found that the CHS mutant genotypes of *Ipomea purpurea* suffered from intense herbivorous damage and was seriously infected by the pathogenic *Rhizoctonia solani* [18]. Various research results have indicated the CHS in plant resistance appears to be inevitable. Different factors variedly caused the expression of CHS, which in turn promoted the production of flavonoids and other related secondary metabolites, which play a key role in plant resistance as antibiotics or ingestion inhibitors. Although some CHS genes have been reported in *Arabidopsis thaliana* and other studied plants, research on the cassava CHS genes is limited, with studies primarily focusing on their role in drought resistance [24].

Cassava (*M. esculenta* Crantz, Euphorbiaceae) is widely planted throughout tropical Africa, Asia and Americas [25]. It is one of the most important root tuber crops for energy in the tropics, with high carbohydrate production potential and adaptability to variety environments. However, cassava is susceptible to mites, especially *T. cinnabarinus*, one of the most serious worldwide pest mites affecting cassava that causes serious economic losses [26]. In our previous study, we evaluated eight cassava varieties through long-term (2016–2018) field trials. According to field identification, the resistance grade for cassava genotype XX048 was categorized as mite-resistant. The content of secondary metabolites, such as flavones, tannin and total phenols increased in response to *T. cinnabarinus* feeding [27]. As the enzymes of the CHS family participate in the synthesis of multiple secondary metabolites in plants, we hypothesize that some members of CHS family may be intimately involved in this process. To further understand the mite-resistant mechanism of *T. cinnabarinus* in cassava, we conducted a comprehensive study on the CHS family.

Recently, systematic studies of the CHS family have been conducted in a variety of plant species such as rice [28,29,30], cotton [31], maize [32], *Petunia hybrida* [33], pea [34], eggplant [35], Antarctic moss [36], morning glory [37], tobacco [38], *Cassia alata* [39], soybean [40], turmeric [41] and *Citrus* [42]. However, the CHS gene family of cassava has not been identified and analyzed yet. In the current study, all *MeCHS* family members were identified, characterized and comprehensively analyzed using a previous cassava genome database. It helped to find out the existence of CHS genes, gene characteristics, their phylogeny relationship, conserved motifs location, gene exon/intron structure, chromosomal localization and gene duplication, cis-elements in the promoter regions, protein-protein interaction (PPI) network, tissue specific expression and verification the expression under *T. cinnabarinus* infection. Previous studies have shown that in most plants, chalcone synthetase genes generally consisted a family of three to twelve members [33,43,44,45,46,47]. This research provides the first evidence of the CHS genes family in cassava, with a comprehensive analysis of 39 identified CHS genes. On the basis of this information, it is possible to characterize unidentified CHS genes from cassava genome in near future, which might serve to elucidate the molecular mechanisms underlying mite-resistance responses in cassava or other plants.

## 2. Materials and Methods

### 2.1. Identification of CHS Genes in Cassava

For the sake of identify the CHS genes, the whole-genome annotations of cassava in the latest version (*Manihot esculenta* v6.1) were downloaded from Ensembl Plants website (http://plants.ensembl.org/index.html, accessed on 2 July 2021). The annotated gene databases were scanned using HMMER 3.0 (http://hmmer.org/, accessed on 2 July 2021), with the Hidden Markov model (HMM) of Chalcone synthase (CHS) domain (PF02797), which was downloaded from Pfam database (http://pfam.xfam.org/, accessed on 2 July 2021). Download the Chalcone synthase (CHS) domain PF02797 from Pfam (http://pfam.xfam.org/, accessed on 8 May 2021). By using the HMM algorithm and setting the E-value < 1 × 10^−×^, the potential CHS genes in cassava genome were searched by hmmsearch (http://hmmer.org/, accessed on 6 May 2021) to construct cassava-specific CHS HMM. The cassava-specific CHS HMM was used to align all protein sequences with an Evalue lower than 1 × 10^−5^ [48,49,50]. In order to avoid any non-specific sequences outside the CHS cluster, we used *A. thaliana* CHS and *Populus trichocarpa* CHS genes as all queries to explore cassava database with default parameters. For this, we used the Pfam database and conservative Domain database (CDD, https://www.ncbi.nlm.nih.gov/Structure/cdd/wrpsb.cgi, accessed on 6 May 2021). Only the total length of the CHS domain sequences was selected in selecting the *MeCHS* genes for subsequent analysis.

### 2.2. Characteristics of CHS Genes

To characterize and align *MeCHS* sequences, ProtParam (http://web.expasy.org/prot-param/, accessed on 2 February 2021) was used to predict the physical and chemical characteristics of *MeCHS* protein, including the length of protein, molecular weight (Mw), predicted theoretical isoelectric point (pI)), instability index, aliphatic index and grand average of hydropathicity of coding proteins.

### 2.3. Phylogenetic, Conserved Motifs and Gene Structure Analysis

The ClustalW algorithm was used in MEGA-7.0.26 (https://www.megasoftware.net/, accessed on 12 February 2021) software with 1000 bootstrap replicates to select multiple sequence alignments of amino acids, and the blank areas were deleted. Table S4 listed the CHS genes used in *A. thaliana* and *P. trichocarpa*. An unrooted phylogenetic tree was constructed via the neighbor-joining (NJ) approach method. Based on the well-established division in *A. thaliana*, the *MeCHS*s were further classified into various subcategories.

The MEME program (http://meme-suite.org/tools/meme, accessed on 1 February 2022) was used to identify the conserved motifs. All the other default parameters were normal, except for the maximum number was set to 20. The gene structure (exon-intron) of *MeCHS* was visualized via the Gene Structure Display Server (GSDS) 2.0 (http://gsds.cbi.pku.edu.cn/, accessed on 1 February 2021) program.

### 2.4. Chromosomal Localization and Gene Duplication Analysis

MapGene2Chromosome (MG2C) v2.0 program (http://mg2c.iask.in/mg2c_v2.0/, accessed on 2 February 2021) mapped every CHS gene with the chromosomal locations, and it was based on the genome annotations of cassava database (http://www.phytozome.net/cassava, accessed on 2 February 2021). Using the CDS sequence of cassava CHS gene, gene duplication was explored according to the following criteria: (a) Length of aligned sequence covers >75% of longer gene, and (b) similarity of aligned regions >75% [51]. The selected pairs of genes were compared by ClustalW.

### 2.5. Cis-Elements in the Promoter Regions Analysis

Using the online software Plantcare website (http://bioinformatics.psb.ugent.be/webtools/plantcare/html/, accessed on 8 July 2022), the cis-elements of *MeCHS* genes were studied at 1500 bp upstream of the translation initiation site ATG in the promoter region. The Gene Structure Display Server (GSDS) 2.0 (http://gsds.cbi.pku.edu.cn/, accessed on 8 July 2022) was used to map the figure. 

### 2.6. Protein-Protein Interaction (PPI) Network Analysis

To further illustrate the correlation between *MeCHS* genes, we used the interologues of *A. thaliana* to predict PPI networks. The STRING software (Version 11.0) was used to draw the proteins functional interaction network diagram, and the confidence parameter was set as 0.4 [52].

### 2.7. Cultivation of Cassava and Rearing of T. cinnabarinus

The plant material selected in this study was clone ‘Xinxuan 048’ (XX048), which has been proved by previous experiments to have stable mite-resistance and high root starch content, and is one of the cultivars popularized for planting in China. The cultivar was obtained from the cassava research group of the Agricultural College, Guangxi University (Nanning, China). All the stem cuttings were planted in a greenhouse inside a gauze element cages. The growth was observed every day to make sure that no mites or other pests were present. Three-month-old XX048 samples were divided into protected and infested (12 mites per leaf) cassava. Each treatment was divided into three replicates with 12 plants per replicate. Different replicates in different areas were planted of the greenhouse, separated by a mesh. 

For different tissues expression analysis, young leaves, mature leaves, petiole, stems, fibrous roots and tuber were harvested from three healthy plants (of the same three-month-old age, protected cultivar). 

For verification of *MeCHS* gene expression, young leaves protected and infested were selected (of the same three-month-old age) for transcript and protein content analysis. At the same time, GO term analysis was performed based on enrichment to better understand the pathways regulated by XX048 genotype.

Healthy *T. cinnabarinus* adults were selected from leaves in the cassava fields at Agriculture College, Guangxi University. After the *T. cinnabarinus* have been identified under a microscope, raising them on the underside of fresh cassava leaves and careful observation to make sure there were no other impurities. Fresh leaves were changed every two days. Leaves infested by *T. cinnabarinus* were cultured under the conditions of temperature 28 ± 1 °C, relative humidity 70 ± 5%, and photoperiod 14 L:10 D.

### 2.8. RT-PCR and Proteomic Analysis

Total RNA was isolated from each sample by using the RNA extraction kit (Huayueyang, Beijing, China), cDNA was synthesized by reverse transcription from 1 μg total RNA with the Takara cDNA Synthesis Kit (Dalian, China). Primers were designed for qPCR using primer 5.0 (Table S9). The qPCR was performed using cassava TAF 15b gene as endogenous reference [53]. Total RNA was isolated from different tissues and real time-qPCR was performed on Roche Lightcycler 480 Real-time PCR System (manufacturer: Roche, city: America, producing area: Switzerland) by using SYBR Green PCR reactions Master Mix kit (Vazyme, Nanjing, China) with cassava TAF 15b actin as the endogenous control. Each reaction was performed with three replications. The relative each gene expression level was calculated by the 2^−ΔΔCT^ method, using the T100 Thermal Cycler software (manufacturer: BioRad, city: America, producing area: Singapore) [54].

Goatools (v0.6.5) (https://github.com/tanghaibao/Goatools, accessed on 13 June 2020) with Fisher’s exact test (α = 0.05) was used for Gene Ontology (GO) functional enrichment analysis [55,56].

## 3. Results

### 3.1. Identification of CHS Genes Family from Cassava Genome Resources

A total of thirty-nine non-redundant chalcone synthase genes (*MeCHS*s) were identified and used for subsequent analyses. The gene IDs of the *MeCHS* members are listed in Table S1. All of the identified *MeCHS* genes contained a complete chalcone stilbene synthases C-terminal (PF02797) domain. The details of *MeCHS* genes with their HMM profiles are summarized in Table S2. Physical and chemical characteristics of *MeCHS* genes were analyzed (Table S3). The protein sequence of *MeCHS* genes ranged from 217 (*MeCHS*36) to 536 (*MeCHS*35) amino acid (aa) residues with a molecular weight (MW) of 24.69 to 60.81 kDa. The theoretical isoelectric point (pI) of *MeCHS*s ranged from 4.91 to 9.34, and the average pI was 8.15, implying that most *MeCHS*s were slightly alkaline. With the exception of fourteen *MeCHS* genes (*MeCHS*4, 5, 8, 9, 11, 21, 22, 23, 29, 31, 33, 35, 38 and 39), all the other fifteen *MeCHS* genes were considered stable (Instability index < 40). The content of aliphatic acids in *MeCHS* genes was high, and the aliphatic index ranged from 76.73 to 105.24. Because of the low average hydropathy value (<0.07), most *MeCHS* were predicted to be hydrophilic.

### 3.2. Phylogenetic Relationship of MeCHS Family

To investigate the evolutionary relationship of CHS gene families further, a total number of 120 CHS proteins, comprising 39 from cassava (Table S1), 31 *A. thaliana* and 50 from *P. trichocarpa* (Table S4), were performed to constructed an unrooted phylogenetic tree using MEGA-7.0.26 software.

**Figure 1:**
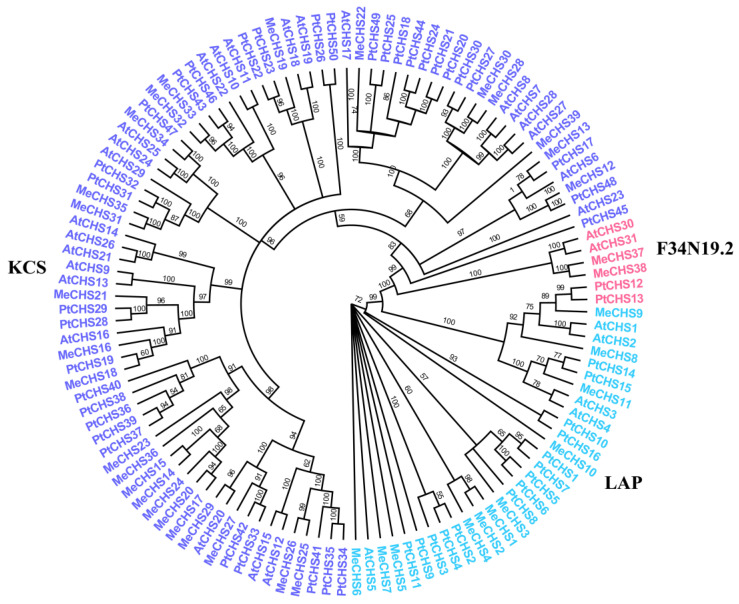
Phylogenetic analysis of CHS genes. Subfamilies are indicated by different colors.

**Figure 2:**
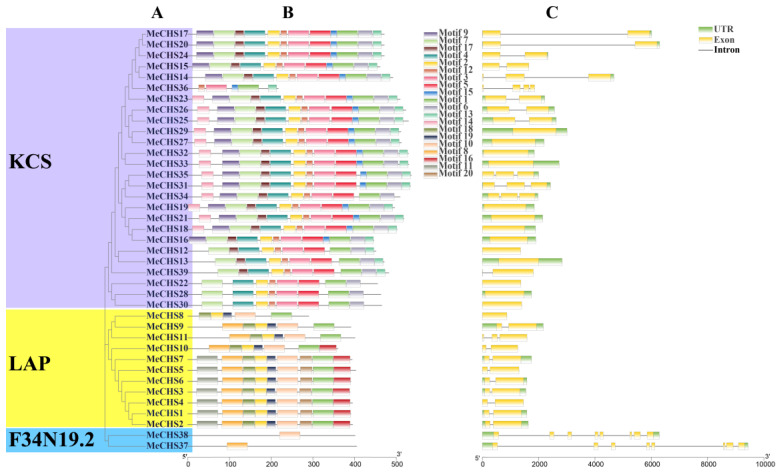
Distribution of conserved motifs and structures of *MeCHS* genes. (**A**) A phylogenetic tree is constructed by neighbor-joining method using *Me*ga-7.0.26 software. (**B**) Conserved motifs distribution of CHS genes. The different motifs are represented by different colors for motifs 1–20, and the structural features of the 20 motifs are listed in Table S5. (**C**) Structures of the 39 *MeCHS* genes. The exons and introns are represented by yellow boxes and black lines, respectively. Green boxes represent the untranslated regions (UTRs) of gene sequences.

All the 120 CHS full-length proteins sequences were clearly clustered into three categories with a bootstrap value of 100%, which could be further classified into KCS, LAP and F34N19.2 subgroups (Figure 1 and Figure 2A). Among them, F34N19.2 subgroup had the least *MeCHS*s (2), KCS subgroup had the most *MeCHS*s (26), and LAP subgroup had an intermediate amount of *MeCHS*s (11). 

### 3.3. Conserved Motifs and Gene Structure Analysis

The diversity of *MeCHS* genes is important for better understanding the characteristics and functions of this gene family in cassava. Evolutionary mechanisms, conserved motifs and gene intron/exon distribution were found in 39 *MeCHS* protein sequence. To further analyze the motifs of the *MeCHS* genes, the maximum number was set to 20 motifs using MEME 5.5.5 software (http://meme-suite.org/tools/meme, accessed on 13 June 2020). The MeMe program identified 20 conserved motifs. The length of the conserved motifs varied from 15 to 50 amino acids, and the conserved motifs features of *MeCHS*s are listed in Table S5. As shown in Figure 2B, motifs from the same subfamily exhibited similar patterns (e.g., *MeCHS*1–11), but there are also differences in motif composition among the three groups. Results indicated that there was total 34 genes which contained three motifs (Motif 2, 13, 16), and the *MeCHS*1–7 genes of LAP all possessed the same 9 motifs. Among the identified motifs, some were specific to special *MeCHS* subfamily. Like 19th motif contain only one member of LAP while motif 17 and 15 was only present in KCS subfamily. These findings provided additional evidence to support specific evolutionary characteristics of the cassava CHS gene family. Moreover, motifs specific to each *MeCHS* group may be considered to have special functions for *MeCHS* proteins.

The diversity of gene structure in polygenic families was an important part of the evolution research. For the purpose, this study conducted a comprehensive structural analysis of *MeCHS* genes by constructing exon-intron map (Figure 2C). A variation in the composition of introns number was found among 39 *MeCHS* members that varied from 0 to 7. Among the 39 *MeCHS* genes, 14 had no introns, 17 had only one intron, 6 contained 2–3 introns, and 2 had seven introns. Additionally, there were similarities in the conserved protein motifs and intron number within the same subfamily of the phylogenetic tree. For example, subfamily KCS had 0–3 introns, LAP had 0–2 introns and F34N19.2 had 7 introns, respectively. Interestingly, conserved intron-exon motif arrangement were found among most of the subfamilies but some differences were also observed. Quoting as an example, the *MeCHS*20/24 exhibits same number of exons and intron phase with variation in intron length. Perhaps these factors may contribute to the size and structure differences of the identified CHS genes in cassava. This suggested that the difference in intron length was one of the reasons for the differences in *MeCHS* genes.

### 3.4. Chromosomal Localization Analysis

**Figure 3:**
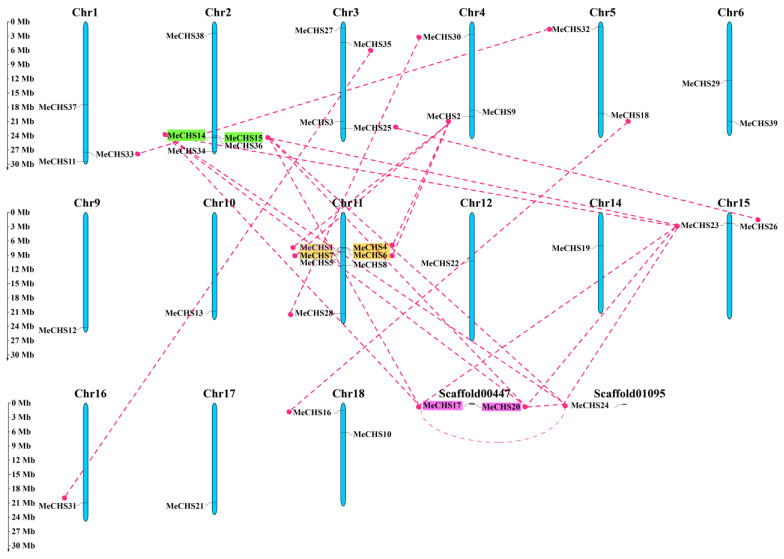
Chromosomal locations of CHS genes in the cassava genome. The duplicated *MeCHS* genes are connected with the pink lines. The tandem duplicated genes are represented by different color backgrounds.

The chromosomal localization map was constructed to specify the corresponding chromosome orientation of *MeCHS*s (Figure 3). Results indicated that the all the *MeCHS*s were unevenly distributed on 15 of the 18 cassava chromosomes (expect Chr7, Chr8, Chr13), Scaffold00447 and Scaffold01095. Chr2 contained the most *MeCHS* genes (*n* = 4; 10.26%), followed by Chr1 (*n* = 3; 7.7%), and then Chr3, Chr4, Chr6, Chr11, Chr15 and Chr18, each of which had two members. There is only one *MeCHS* member on the rest of the chromosome (Chr5, Chr9, Chr10, Chr12, Chr14, Chr16 and Chr17). In particular, *MeCHS*17 and *MeCHS*20, *MeCHS*24 were existed in Scaffold00447 and Scaffold01095, respectively. Notably, more than half of the *MeCHS* genes (*n* = 21, 53.85%) were concentrated at either end of the chromosomes. The *Arabidopsis* and apple genomes also contain such prejudiced distribution pattern [57].

Duplication in the genome may contribute to the expansion of gene families and the development of variants for new functions [58,59]. In an organism, gene duplication occurred through segmental, tandem or whole genome. As shown in Figure 3, we found segmental and tandem duplication event in *MeCHS* genes. Tandem duplication genes *MeCHS*14/15 was located on Chr2, and two other duplication repeat genes (*MeCHS*1/4/6/7 and *MeCHS*17/20) were located on Chr11 and Scaffold00447, respectively. In addition, 25 pairs of *MeCHS* genes were identified as segmental duplication, and the duplication screening results are listed in Table S6.

### 3.5. Promoters Cis-Elements Analysis 

**Figure 4:**
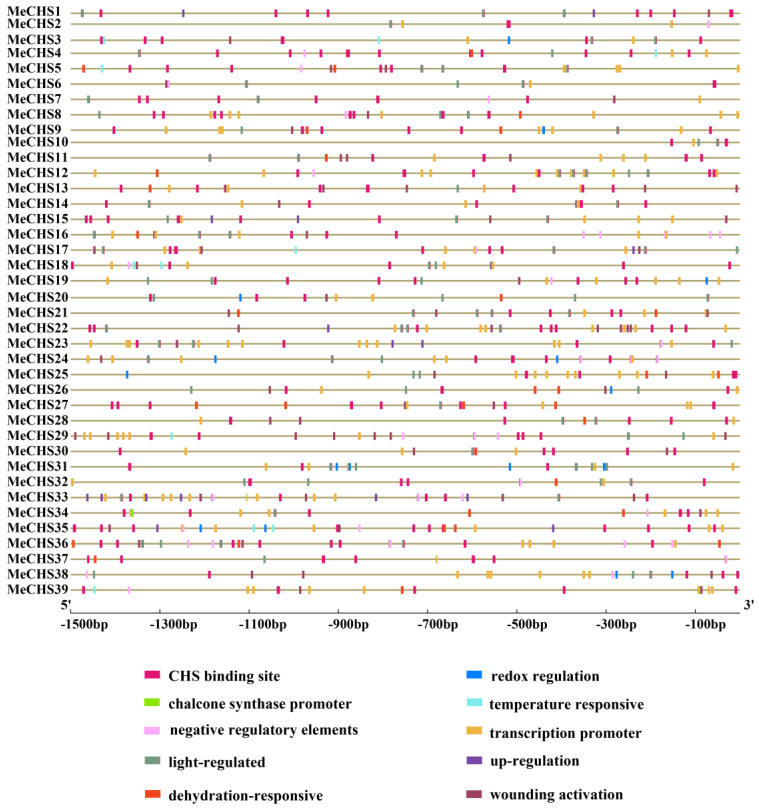
Prediction of cis-elements in the promoter regions of *MeCHS*s. The number at the bottom represents the distance to the translation start codon, ATG.

In order to clearly understand the potential regulatory mechanism of *MeCHS*s, cis-elements were investigated in the promoter region (1500 bp upstream of the *MeCHS*s translation initiation site, ATG). We selected 10 functional predicted cis-elements in the promoter region of *MeCHS* genes, which are listed in Table S7. The promoter sequences of these probable stress-responsive *MeCHS* genes were analyzed in the Plantcare database for the possible predominant occurrence of cis-regulatory elements in stress response (Figure 4). These cis-elements could be divided into five groups: (1) elements related to chalcone synthase (CHS); (2) wounding gene activation elements; (3) associated with light-regulated elements; (4) stress, such as low-temperature; (5) related to plant disease resistance. These findings suggest that the gene expression patterns of *MeCHS* may be regulated by various cis-elements within the promoter, such as wounding activation, low temperature stress, etc.

### 3.6. Protein-Protein Interaction (PPI) Network Analysis

**Figure 5:**
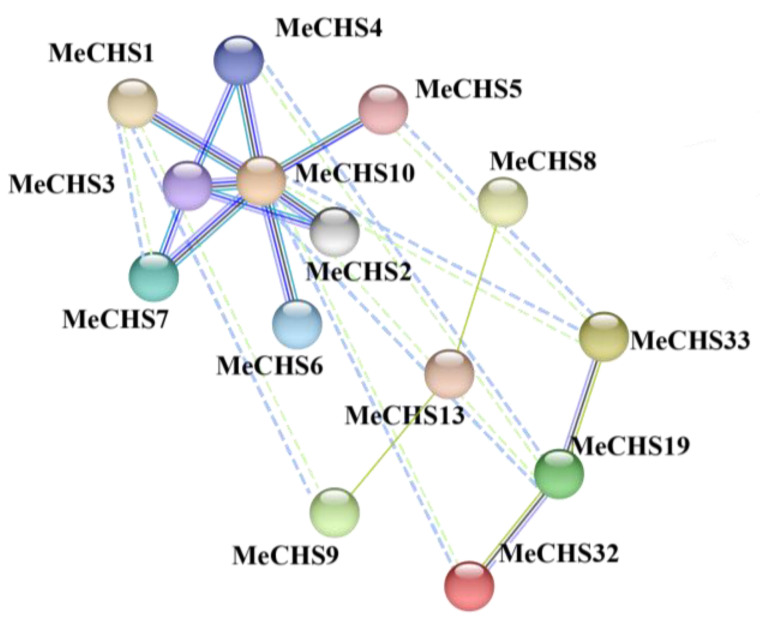
Protein-protein interaction (PPI) network analysis of the *MeCHSs*.

We used *A. thaliana* orthologues proteins to construct a PPI network by STRING software (version 12.0) to further understand the association of the *MeCHS* proteins. For reliability, only 14 *MeCHS* proteins with the highest combined score were selected (Figure 5). The protein-protein interaction (PPI) network analysis of *MeCHS* genes were listed in Table S8. In particular, the *MeCHS*1 and *MeCHS*10 protein are more closely related to other family members. These protein interaction networks might provide relevant clues to understanding the function of unknown genes.

### 3.7. Analysis of MeCHS Gene Expression in Different Tissues

**Figure 6:**
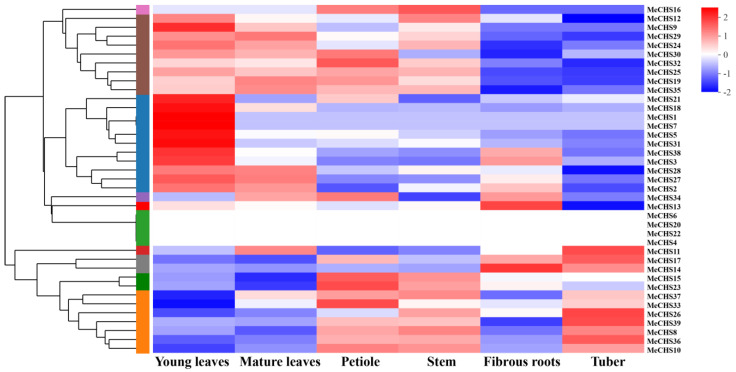
Differential tissues expression of *MeCHS* genes (with no infested by *T. cinnabarinus*). On the left are the tree diagram of gene cluster and the module diagram of subcluster, and on the right are the names of the genes. The color in the figure represents the expression value of this gene after standardized treatment in each sample. Colors from red to blue indicate high to low expressions level. The numbers in the upper right color represent trends in gene expression levels. Differential tissues expression of *MeCHS* genes were listed in Table S10.

A large amount of evidence shows that the role of CHS genes in plant growth and development cannot be ignored. To understand the physiological function of *MeCHS*s better, RT-qPCR was used to detect the transcription level of *MeCHS* gene in different tissues such as young leaves, mature leaves, petiole, stems, fibrous roots and tuber of XX048 in 3-month-old protected samples. The sequences of primers used in RT-qPCR were listed in Table S9. And a heat map of the hierarchical clustering was generated to show the expression profiles of the *MeCHS*s in different tissues (Figure 6). The expression of four *MeCHS* genes (*MeCHS*6, 20, 22, 4) was not detected in any of the tissues analyzed, which may be due to differences in spatiotemporal expression patterns. The expression of *MeCHS*s revealed a tissue- and organ-specific pattern, which showed a relatively high expression level in young leaves and mature leaves, but they had a lower expression in petiole, stems, fibrous roots and tuber. Specifically, *MeCHS*21, 18, 1, 7, 5, 31, 38, 3, 28, 27 and 2 were highly detected in young leaves, while the expression of the following genes *MeCHS*15, 23, 37, 33, 26, 39, 8, 36 and 10 were weak expression. In general, the expression level of *MeCHS* genes in young leaves was higher than in other tissues (among 39 genes, 21 were highly expressed), but differences are also observed in the same tissue of CHS genes. The differential expression of these *MeCHS* genes that were highly articulated in plant tissue may have important function in plant development processes, which needs to be further verified.

### 3.8. Verification of MeCHS Gene Expression

To further investigate the potential roles of *MeCHS* in *T. cinnabarinus*, nine genes in leaves of XX048 protected and infested (12 mites per leaf) with *T. cinnabarinus* treatments were selected for transcript and protein content analysis to confirm the correlation of mRNAs and their corresponding proteins expression profiles. 

**Figure 7:**
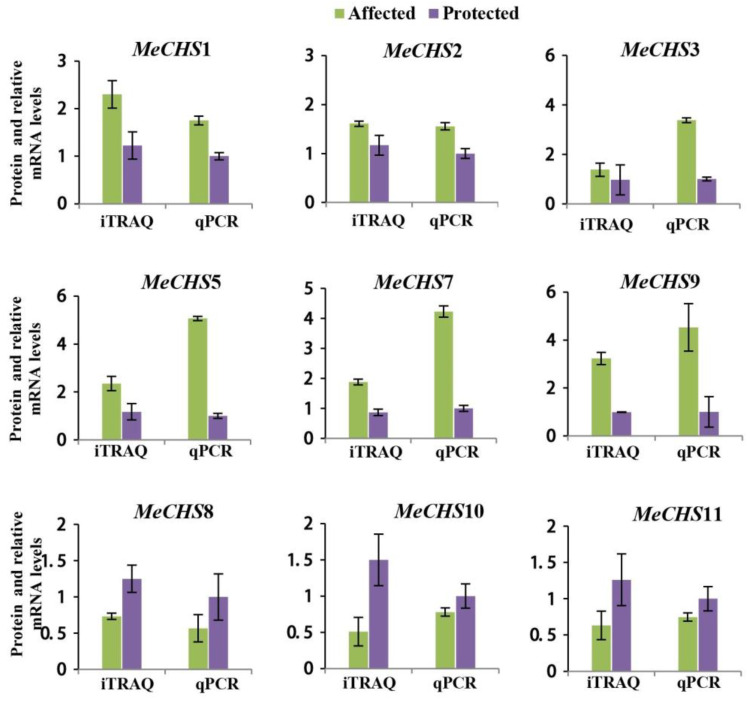
Relative expressions of 9 genes at the protein and mRNA levels.

The qPCR results showed the expression profiles of the 9 genes (*MeCHS*1, *MeCHS*2, *MeCHS*3, *MeCHS*7, *MeCHS*31, *MeCHS*38, *MeCHS*33, *MeCHS*37, *MeCHS*39) were in agreement with the protein content data (Figure 7). The expression profiles of qPCR and protein content were consistent, indicating that *MeCHS* genes played different roles in the process of resisting *T. cinnabarinus*, which might be realized through up-regulation or down-regulation of these genes.

### 3.9. Gene Ontology (GO) Enrichment Analysis of MeCHS Genes

**Figure 8:**
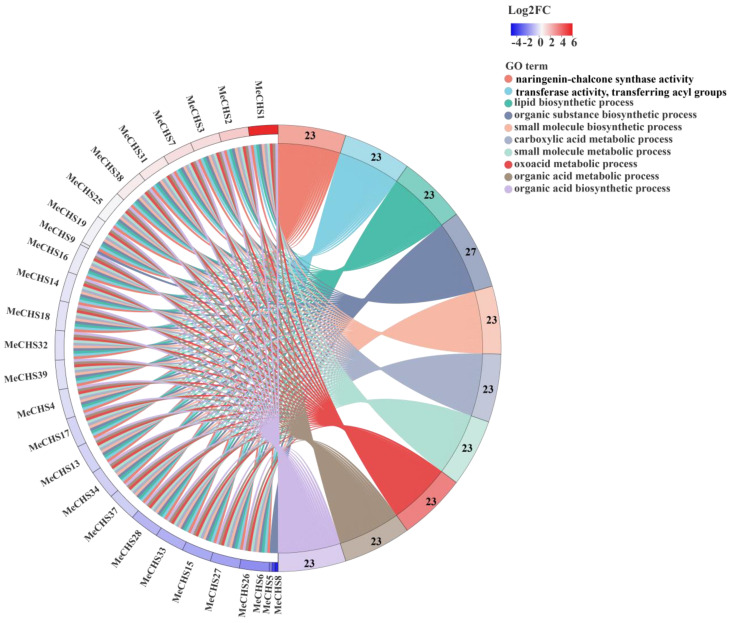
GO term functional enrichment chord diagram. On the left are the *MeCHS* genes, which is arranged from large to small in proportion to the infected and protected expression log2FC value. On the right is the GO Term information on significant enrichment. The image was generated by the software Goatools (Version 0.6.5).

GO term analysis of the *MeCHS* genes was performed based on enrichment to gain deeper insight into the pathways regulated by XX048 genotype (Figure 8). The GO enrichment analysis of *MeCHS* genes was carried out by using software Goatools to classification the functions with Fisher exact test. In order to control the false positives in the calculation process, BH method is used to adjuste the *P* value (adjusted *p* value). When the adjusted *p* value < 0.05, the GO function was considered to be significantly enriched. In this study, CHS genes of affected and protected leaves were analyzed according to log2 fold change (log2 FC), the details GO terms of *MeCHS* genes are listed in Table S12. The value of log2FC indicates the differential multiple of the up-regulated gene expression of affected and protected. As showed in Figure 8, *MeCHS*1, *MeCHS*2 and *MeCHS*3 had the largest log2FC among all *MeCHS* genes, which were 5.64, 1.68 and 1.24, respectively. On the other hand, *MeCHS* genes involved in organic substance biosynthetic process were significantly enriched in all GO terms (number of genes: 27). In addition, the activity of naringenin-chalcone synthase, which related to CHS were also significantly enriched in GO terms (number of genes: 23). Therefore, these results suggested that *MeCHS* in XX048 were involved in a variety of biological processes under the infection of *T. cinnabarinus*. 

## 4. Discussion

In recent years, as an attempt to understand the CHS genes, genome-wide identification of CHS gene family has been carried out in rice (*Oryza sativa*, 31 members) [28,29,30], cotton (*Gossypium barbadense*, 20 members) [31], maize (*Zea mays*, 14 members) [32], *Petunia hybrida* (8 members) [33], pea (*Pisum sativum*, 7 members) [34], eggplant (*Solanum melongena*, 7 members) [35], Antarctic moss (*Pohlia nutans*, 6 members) [36], morning glory (6 members) [37], tobacco (*Nicotiana tabacum*, 5 members) [15,38], *Cassia alata* (3 members) [39], soybean (*Glycine max*, 3 members) [40], turmeric (*Curcuma longa* Linn., 3 members) [41] and *Citrus* (2 members) [42]. However, the diversity (in varied aspects) and characterization of the CHSs gene family in cassava have not been comprehensively investigated. Our research identified a total of 39 CHS genes (*MeCHS*1–39) in cassava genome resources, which is one of the most CHS family members identified in one plant to date. 

On the basis of the open genomic information of cassava, we investigated the CHS gene family. We comprehensively analyzed their physical and chemical characteristics, including protein length, molecular weight, theoretical isoelectric point, instability index, aliphatic index and grand average of hydropathicity. In order to further understand the *MeCHS* gene family, we analyzed their phylogenetic classification, conserved motifs and exon-intron placement, chromosomal localization, cis-elements, PPI network, different tissues expression and responses to *T. cinnabarinus*. This study is the first thoroughly analyzed the evolution of the CHS family in cassava, and the resulting information certainly useful for providing further evidence to hypothesize about the potential functions of unknown genes.

Phylogenetic analysis of the 120 CHSs members (comprising 39 from cassava, 31 *A. thaliana* and 50 from *P. trichocarpa*) were divided into three subgroups according to their sequence homology and classification from *A. thaliana* [60]. All three subgroups (KCS, LAP and F34N19.2) included *A. thaliana* genes, implying that the CHS genes have been conserved over long evolutionary periods. During the development of *A. thaliana*, leucine aminopeptidase (LAP), protein can be detected at different developmental stages and in different organs after treatment with phytohormones or by wounding, with there being slight differences in the LAP accumulation and organ-specificity. In other words, LAP is present during all stages of development in *A. thaliana* [61]. In general, genes grouped into the same subclasses have similar evolutionary characteristics and acquired the same expression pattern. Eleven cassava genes (*MeCHS*1–11) were clustered into LAP subclass, and analysis of the conserved motifs further confirmed the classification of the *MeCHS* family. At least five identical conserved motifs have been found in the LAP subclass, which may play an important role in its function. It is worth noting that *MeCHS*1 and *MeCHS*10 in the LAP subclass are shown to be more closely related to other family members in the PPI network. These results suggest that genes/proteins in the LAP subfamily deserve further investigation of their function.

*MeCHS*s were distributed on fifteen cassava chromosomes, Scaffold00447 and Scaffold 01095, which had extensive variation in protein length, molecular weight, theoretical isoelectric point, instability index, aliphatic index and grand average of hydropathicity. These differences in CHS genes might be linked to gene duplication events or genome size [62]. In the current study, a total of three tandem duplicated *MeCHS* genes and twenty-five pairs of segmental duplicated *MeCHS*s were detected. Due to the large number of segmental duplication, they appear to contribute more to cassava CHSs expansion than tandem duplication. The *MeCHS*s located on almost all chromosomes except Chr7, Chr8, Chr13 and were unevenly distributed. Remarkably, more than half of the *MeCHS* genes were concentrated at either end of the chromosomes (21 members). 

Previous studies have shown that the CHS genes in most plants contained one intron at a conserved location, except *Antirrhinum majus* (*Plantaginaceae*) which has two introns [63]. However, some recent investigations of characterization of CHS gene families from *Alpinia calcarata* [64], *Physcomitrella patens* [65], *Rheum emodi* [66], and *Polygonum cuspidatum* [67] have demonstrated CHS genes either as intronless or with more than two introns. Our study demonstrates that among the 39 *MeCHS* genes of cassava, 17 had only 1 intron (43.59%), 14 had no intron (35.90%), 6 contained 2–3 introns (15.38%), and 2 had 7 introns (5.13%). The number of introns presence in the exons of these characterized genes may have important implications for the evolution of the CHS gene family in the plant family. For instance, an unusual intron was found in the second exon of a type III polyketide synthase gene of *A. calcarata* Rosc, representing an insight into information regarding the distribution of novel genes in Zingiberaceae. 

The CHS binding site response clusters were found in all the 39 CHS genes of cassava, and they belonged to different stress-response clusters. Several abiotic and biotic stress-responsive cis-elements have been identified. Among the 39 promoters studied, 33 had the wounding activation element denoting the possible biotic response regulation, 22 had dehydration-responsive element, 8 had low-temperature responsive element. Besides, we also found light-regulated, plant disease resistance elements. The importance of interaction between cis-elements in stress-responsive transcription has been demonstrated in several studies [68,69], and cis-element are indispensable for stress-responsive transcription [70]. Therefore, multiple stress-responsive elements are present in these CHS genes. As XX048 is relatively mite-resistant crop, these putative CHS proteins might play an important function in conferring mite resistance. In addition to investigating the LAP subclass in a phylogenetic and PPI network, it is necessary to further confirm the important role of *MeCHS* gene in mite resistance.

The key role of CHS genes in controlling flavonoid biosynthesis pathways has been well demonstrated in many plant species [71,72,73]. The formation of resistance in plants is a complex process that associated with the secondary metabolites produced by plants, such as flavonoids. In our study, most of the LAP subclass like *MeCHS*1, *MeCHS*2, *MeCHS*3, *MeCHS*7, *MeCHS*31 and *MeCHS*38 had high expression level in young leaves, which may have affected the cassava resistant to mites through the production of flavonoids by cell expansion or division in cassava. The RT-qPCR and proteomic analysis of the expression of LAP subclass member in protected and affected plants showed that they play a specific role in cassava leaves, suggesting that *MeCHS*s played an important role in response to *T. cinnabarinus*.

This comprehensive study provides a basis for further investigation of cassava CHS genes and could also has potential value for the genetic improvement of cassava and other related species.

## 5. Conclusions

The diversity in CHS gene family structure specifies their vast range of functions. Until recently, the *MeCHS* genes regulatory system during plant growth and development was poorly understood. Our study is the first comprehensive characterization of CHS genes in cassava and identified 39 *MeCHS* gene family members from the cassava genome. Through gene evolution and selection analysis, the diversity and extension of the family members were clarified. These data provide basic information about the family for future investigation into the CHS-mediated molecular mechanism underlying plant growth, development, and mite resistance. This study could provide reference for functional research and molecular breeding of cassava.

## Data Availability

All the proteomic analysis mass spectrometry data have been deposited into the iProX (https://www.iprox.org, accessed on 1 January 2022) with the identifer IPX0001727000.

## Supplementary Materials

The following supporting information can be downloaded at https://www.mdpi.com/article/10.3390/genes15030336/s1: Table S1. List of the CHSs sequences in cassava. Table S2. A catalog of *MeCHS* genes with their HMM profiles. Table S3. Physical and chemical characteristics of *MeCHS* genes. Table S4. CHS genes used in *A. thaliana* and *P. trichocarpa*. Table S5. Conserved motifs of *MeCHS*s. Table S6. Duplication screening results of *MeCHS* genes. Table S7. Cis-elements in the promoter regions of *MeCHS* genes. Table S8. Protein-protein interaction (PPI) network analysis of *MeCHS* genes. Table S9. Sequences of primers used in RT-qPCR. Table S10. Differential tissues expression of *MeCHS* genes. Table S11. Relative expressions of 9 genes at the protein and mRNA levels. Table S12. GO term analysis of *MeCHS* genes.

## Abbreviations

Aa: Amino acid; Chr: Chromosome.
